# A Comparison Study of Machine Learning Based Algorithms for Fatigue Crack Growth Calculation

**DOI:** 10.3390/ma10050543

**Published:** 2017-05-18

**Authors:** Hongxun Wang, Weifang Zhang, Fuqiang Sun, Wei Zhang

**Affiliations:** School of Reliability and Systems Engineering, Beihang University, Haidian District, Beijing 100191, China; wanghongxun@buaa.edu.cn (H.W.); zhangweifang@buaa.edu.cn (W.Z.); sunfuqiang@buaa.edu.cn (F.S.)

**Keywords:** fatigue crack growth, machine learning algorithms, fatigue life prediction, extreme learning machine (ELM), stress ratio

## Abstract

The relationships between the fatigue crack growth rate (da/dN) and stress intensity factor range (ΔK) are not always linear even in the Paris region. The stress ratio effects on fatigue crack growth rate are diverse in different materials. However, most existing fatigue crack growth models cannot handle these nonlinearities appropriately. The machine learning method provides a flexible approach to the modeling of fatigue crack growth because of its excellent nonlinear approximation and multivariable learning ability. In this paper, a fatigue crack growth calculation method is proposed based on three different machine learning algorithms (MLAs): extreme learning machine (ELM), radial basis function network (RBFN) and genetic algorithms optimized back propagation network (GABP). The MLA based method is validated using testing data of different materials. The three MLAs are compared with each other as well as the classical two-parameter model ($K^{*}$ approach). The results show that the predictions of MLAs are superior to those of $K^{*}$ approach in accuracy and effectiveness, and the ELM based algorithms show overall the best agreement with the experimental data out of the three MLAs, for its global optimization and extrapolation ability.

## 1. Introduction

As the damage tolerance concept has been widely accepted and applied in the aerospace engineering, it becomes necessary and important to calculate the fatigue crack growth. As is known, da/dN−ΔK curve in the log-log coordinate has three characteristic regions, which are named threshold region (region I), Paris region (region II) and high ΔK region (region III). It can be observed in extensive experimental results that the relationships between fatigue crack growth rate (da/dN) and stress intensity factor range (ΔK) are not linear even in the Paris region [1,2,3,4]. Furthermore, the stress ratio effects on da/dN−ΔK curves are diverse in different materials. For some materials, such as 2324-T39 aluminum alloy [5], 7055-T7511 aluminum alloy [5] and Ti10V2Fe3Al titanium alloy [6], the stress ratio effects are almost the same in the three regions. In other words, the da/dN−ΔK curves with different stress ratios are approximately “parallel”. However, for some other materials, the stress ratio effects on the da/dN−ΔK curves are quite different in the three regions, which means it depends on ΔK, such as spheroidal graphite cast iron [3] and ADB610 steel [7]. Therefore, the relationship between da/dN and ΔK is of complex nonlinearity.

To fit the nonlinear relationship between da/dN and the applied loading, many explicit exponential models based on the fundamental theory of linear elastic fracture mechanics (LEFM) are proposed. In the 1960s, Paris and Erdogan proposed the model [8], which has become the most famous and fundamental fracture mechanics based equation for fatigue life prediction. Paris’ law only can be applied to handle the da/dN in the Paris region, and it does not consider the nonlinearity in the Paris region. Besides, Paris’ law is just a preliminary model without considering the effects of other parameter factors, such as the stress ratio (*R*), the threshold stress intensity factor range $\left(\Delta K_{th}\right)$ and the critical stress intensity factor $\left(K_{c}\right)$. Therefore, researchers proposed many kinds of modified models to consider different nonlinear factors. Forman [9] included *R* and $K_{c}$ directly to make the model can be effectively applied to the Paris region and the high ΔK region. Forman’s model does not consider the effect of $\Delta K_{th}$ and still cannot be used for the threshold region. Additionally, Forman’s model does not change the nature that it ignores the nonlinearity in the Paris region. Priddle’s model [10] can be applied to the three regions by taking $\Delta K_{th}$ and $K_{c}$ into account, and it can fit the Paris region with nonlinearity. However, it does not consider the stress ratio effects.

Based on the mechanism of fatigue crack growth, Elber [11,12] proposed an effective stress intensity factor range $\left(\Delta K_{eff}\right)\;$to involve the effect of crack closure. The $\Delta K_{eff}$ and ΔK can be connected by the parameter U which is related to stress ratios. Theoretically, Elber’s model not only includes the stress ratio effects, but also can be applied to the three regions. Actually, McClung [13] proved that the U changes with $K_{max}\;$in different tendencies in the three regions. No sole equation can cover the crack closure characteristics in the three regions. Therefore, this model is only applicable in a certain range of *R*.

Further studies by Kujawski [14], Donald [15] and Sandananda [16] indicate that crack growth rate is determined by both ΔK and $K_{max}$. Based on this discovery, Dinda and Kujawski [17] suggested the two-parameter model where the fatigue crack driving force parameter $K^{*}$ was proposed instead of $\Delta K_{eff}$. In this model, the $K^{*}$ and the fatigue crack growth rate can be expressed as

(1) $$K^{*}={\left(K_{max}\right)}^{\alpha }{\left(\Delta K^{+}\right)}^{1-\alpha },$$

(2) $$da/dN=f\left(K^{*}\right),$$

where *α* is correlation parameter, and $\Delta K^{+}$ is the positive part of applied ΔK. By involving $K_{max}$ and $\Delta K^{+}$, the $K^{*}$ approach excellently correlates the stress ratio effects and the fatigue crack growth rate. Furthermore, it is applicable to the three regions with nonlinearity even in the Paris region, and it has no limitation on *R* range, which makes it superior to other analytic formulas. However, $K^{*}$ approach has difficulty in accurately solving *α* for different materials and cannot effectively handle the materials whose stress ratio effects on the da/dN−ΔK curves are diverse in the three regions.

Many researchers have been making efforts to find an explicit mathematic formula to match the nonlinearities between the crack growth rate and the driving force parameters by employing more related parameters, such as *R*, $\Delta K_{th}$, $K_{c}$ and $\Delta K_{eff}$. However, the current formulas are not flexible and accurate enough to handle the various situations appropriately. Therefore, the fatigue crack growth should be a nonlinear and multivariable problem, and it is hard to find a generalized and explicit function to account for the effects of all the factors appropriately.

To overcome the shortcomings of classical models in fatigue crack growth estimation, more and more interdisciplinary methods are introduced. The numerical approach and the machine learning method are the most widely used which have been demonstrated to be effective. Additionally, the combination of numerical approach and machine learning algorithms (MLA) is also an evaluable investigation direction. For example, the knowledge-based neural network (KBNN) associates with the finite element method and optimization algorithms [18]. The numerical approach is usually used to simulate the fatigue crack growth process together with the classical models. Bhattacharya et al. [19] applied the extended finite element method (XFEM) to the simulation of the interface fatigue crack growth in the bi-layered material. Hu et al. [20] predicted the fatigue crack growth under variable amplitude loading by utilizing the singular finite element which includes the fracture process zone located in front of the crack tip by using cohesive zone model. The machine learning method provides an alternative and flexible approach to the modeling of the fatigue crack growth rate because of its excellent nonlinear approximation and multivariable learning ability, which make it an advanced and promising method [21,22,23]. Machine learning is a series of algorithms used in data-driven system, such as support vector machine (SVM), genetic algorithms, artificial neural network (ANN), fuzzy logic, neural-fuzzy system and particle swarm optimization (PSO) [24], etc. The great learning and generalization ability of MLA make it can model the internal connections and tendencies from complicated or imprecise data. Therefore, the machine learning methods have been used in fatigue domains for different purposes [25,26,27,28,29]. Zio et al. [30] applied the relevance vector machine (RVM) to predict the remaining useful life of a structure. The application compares well to the model-based Bayesian approach of particle filtering. Mohanty et al. [31] successfully used the genetic program for fatigue life prediction of 2024-T3 aluminum alloy. However, this study did not validate the method’s applicability for different materials. Zhang et al. [32] utilized one of the MLAs: the radial basis function network, to model the fatigue crack growth. The method shows pretty good applicability for different aluminum alloys. However, the distinctions of different MLAs in fatigue crack growth calculation are not investigated, and it is difficult to choose which MLA is more applicable to fatigue crack growth.

In this paper, a fatigue crack growth calculation method is proposed based on three MLAs: Extreme learning machine (ELM), radial basis function network (RBFN) and genetic algorithm optimized back propagation network (GABP). The rest paper is organized as following: First, the basic theories of the three MLAs for nonlinear learning are introduced, and the MLA based fatigue crack growth calculation method is established. After that, the three MLAs are validated and compared by using testing data of different materials. The classical $K^{*}$ approach is also employed as a comparison. Finally, some conclusions and future work are discussed.

## 2. Methodology

### 2.1. Machine Learning Algorithms

#### 2.1.1. Genetic Algorithms Optimized Back Propagation Network

Back propagation (BP) network is one of the most widely used MLAs because of its excellent learning ability and flexible structure. Genetic algorithm (GA) is a parallel computing optimization method by simulating the natural genetic mechanism and theory of biological evolution. The weights and thresholds of the BP network should be randomly initialized whenever it is trained, which will lead to the inaccuracy and instability of the training. The GA optimized BP network (GABP) is to use GA to improve the accuracy and stability by optimizing the initial weights and thresholds.

**Figure 1:**
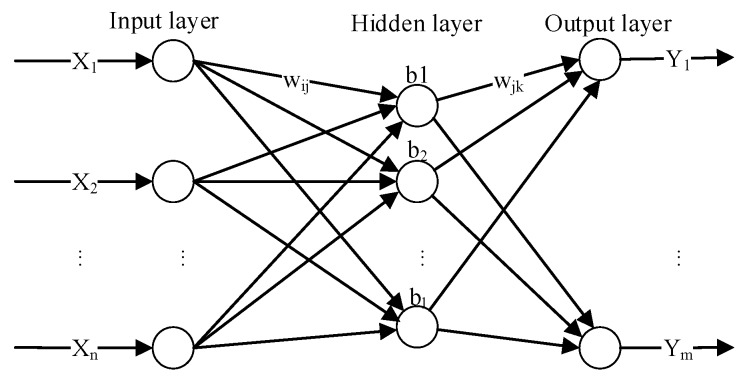
Schematic diagram of back propagation (BP) network.

BP network is the core of multi-layer feed-forward neural networks (FFNNs). The structure of the three layers BP network is shown in Figure 1, where $\left\{X_{1},X_{2},\ldots ,X_{n}\right\}$ and $\left\{Y_{1},Y_{2},\ldots ,Y_{n}\right\}\;$are the inputs and outputs; $w_{ij}$ and $w_{jk}$ are the connection weights between different layers; and $\left\{b_{1},b_{2},\ldots ,b_{l}\right\}\;$are the thresholds of hidden layer. The signals can only transfer forward in the BP network. The BP network utilizes error back propagation algorithms to reduce the training errors.

GA applies the principle of reproduction and survival of the fittest to the populations whose chromosomes (individuals) are encoded by parameters to be optimized. The chromosomes with better fitness are chosen by repeatedly using genetic operations (selection, cross and mutation), until the best chromosome is determined.

**Figure 2:**
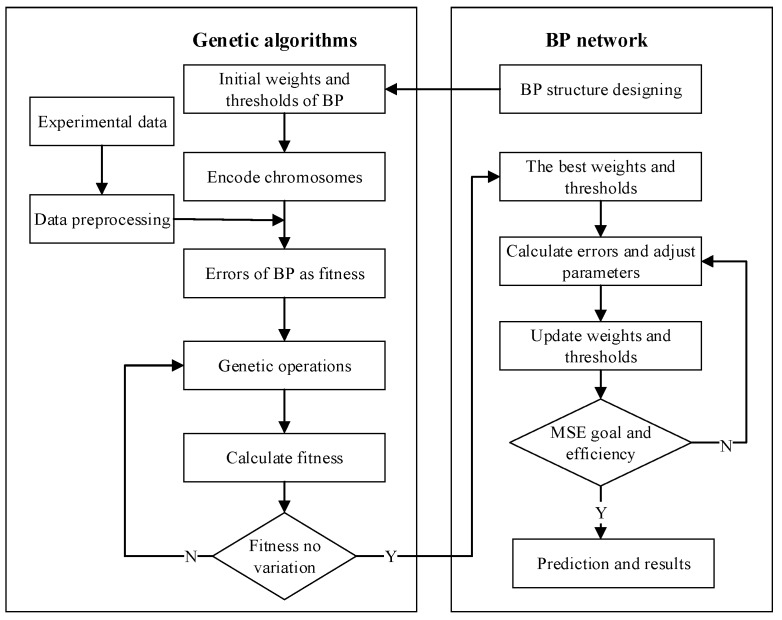
Procedures of genetic algorithms optimized back propagation (GABP) algorithms.

In the GABP, the initial weights and thresholds of BP network are used to encode the chromosomes of GA as shown in Figure 2. The best chromosome determined by GA is assigned to BP network as the initial thresholds and weights. Then the BP network is trained by adjusting the corresponding parameters.

#### 2.1.2. Radial Basis Function Network

Radial basis function network (RBFN) is one of the MLAs using multi-dimensional spatial interpolation technique. The structure of RBFN is similar with the three-layer FFNN as shown in Figure 1. The difference with other FFNNs is that the input layer of RBFN is only used to transfer information without weights variation between the input layer and the hidden layer. Additionally, the RBFN is a partial full-connected network, which improves the shortcoming of easily getting into local minimum in BP network. The neuron number of the hidden layer will be optimized during training. The radial basis function (RBF) of the hidden layer is Gaussian function which is a nonnegative and nonlinear partial response function for its radially symmetric and attenuated characteristics.

The RBFN can use different learning algorithms depending on the different methods for selecting the center of activation function. In this paper, the self-organizing selection center method is utilized. The hidden layer uses nonlinear strategy to tune the parameters of RBFN, while the output layer uses linear optimal strategy to adjust the linear weights. Therefore, the RBFN can be trained quicker than BP network and can handle nonlinear problem with complex mappings [33,34]. It has been demonstrated to be suited for fatigue crack growth under constant and variable amplitude loading [32].

#### 2.1.3. Extreme Learning Machine

Extreme learning machine (ELM) is an emerging MLA, which is a single-hidden layer feed-forward neural network (SLFN) [35]. The structure of ELM is similar with the three-layer FFNN as shown in Figure 1. In the ELM, the layers are fully connected. ELM can randomly generate the connected weights from the input layer to the hidden layer as well as the thresholds in the hidden layer before training. Then the values need not to be iteratively tuned during training process. The only free parameter need to be learned is the weights between the hidden layer and the output layer. Additionally, an infinitely differentiable function must be selected as the activation function of the hidden layer, which is usually sigmoid function. Only by setting the neuron number in the hidden layer, the ELM can reach an optimal generalization bound. Furthermore, ELM overcomes the shortcomings of slow convergence and local minimum problem [36]. Compared with BP network, RBFN and other traditional FFNNs, ELM improves the training rate and tends to attain a global optimum. The advantages of ELM in efficiency stability and generalization performance have been proved by many studies in different domains [37,38,39], which make it a promising approach in machine learning and computational intelligence [36,40,41]. However, there is still no study using ELM for fatigue crack growth.

### 2.2. Design and Training of the Machine Learning Algorithms

**Figure 3:**
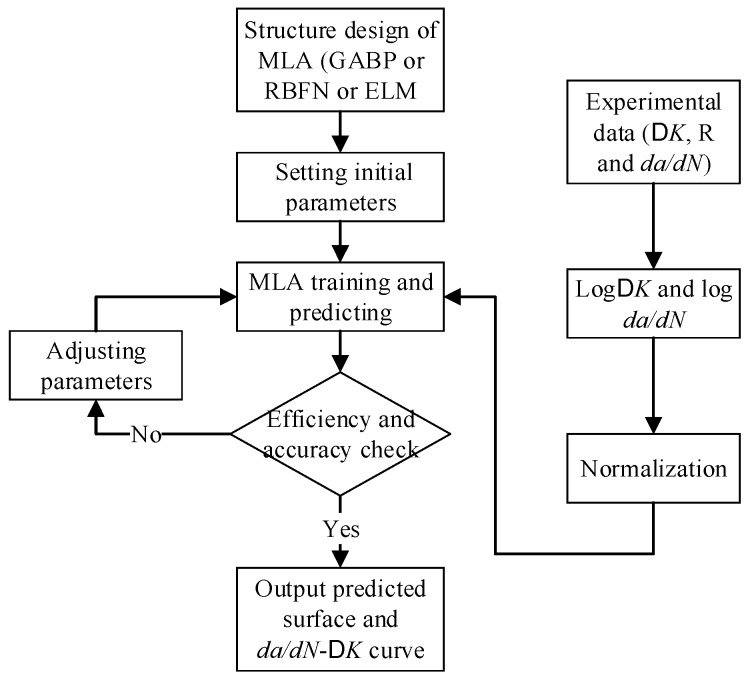
Procedures of designing a well-trained machine learning algorithms (MLA).

The MATLAB R2014 of MathWorks is utilized to design and train the machine learning based algorithms for fatigue crack growth. To develop the well-trained MLAs used for fatigue crack growth, the following procedures should be done as shown in Figure 3. Firstly, the two-input and single-output MLAs (GABP, RBFN and ELM) are established by analyzing the physical driving forces of fatigue crack growth rate under constant amplitude loading. Then the ΔK and *R* are selected as the inputs, and the da/dN is the output. Secondly, the raw experimental data (ΔK, *R* and da/dN), which are acquired from open literature, have to be selected and preprocessed before it can be used in the MLAs. The data preprocessing contains two steps. The first is to take the natural logarithm of da/dN and ΔK. The second is to normalize the logda/dN and logΔK obtained from the first step. After that, the raw experimental data have been transformed into a set of vectors which can be used to train the MLAs. Thirdly, the initial training parameters of the three MLAs are randomly set, and the MLAs are trained. To attain the well-trained MLAs, the accuracy and efficiency of the MLAs should be balanced by comparing the outputs with the experimental data as well as the mean squared error (MSE). The outputs can be optimized by repeatedly adjusting some training parameters. There are seven training parameters for GABP: the maximum generations, the population size, the cross probability, the mutation probability, the maximum training epochs, the learning rate and the MSE goal. The former four are used to optimize the GA and the latter three are used to improve the BP network. Compared with GABP, RBFN has fewer training parameters, which are the expansion speed, the MSE goal and the maximum number of neurons. ELM is the most convenient of the three MLAs, because it just need to adjust the neurons number in the hidden layer to complete training. Finally, the well-trained MLAs output the predicted surface and da/dN−ΔK curves as the following example shows.

**Figure 4:**
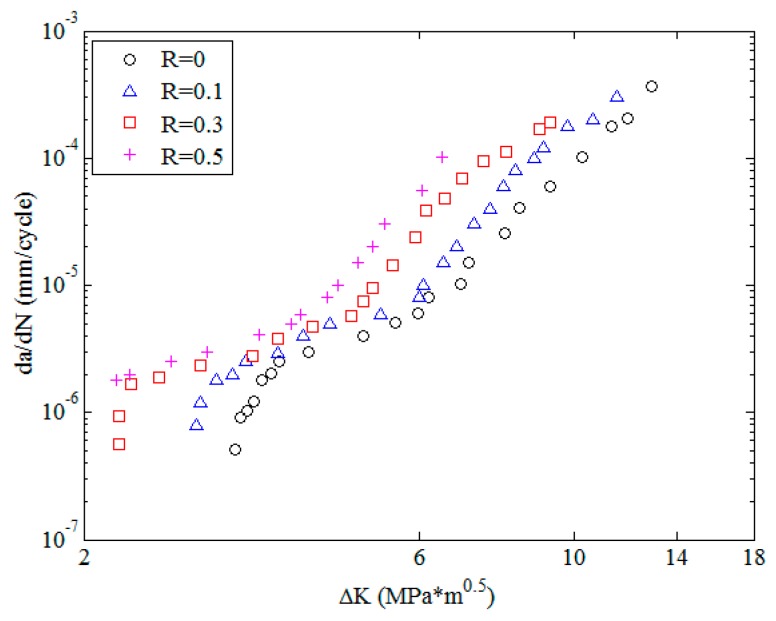
The experimental data of 2024-T351 aluminum alloy in natural logarithm.

To clearly describe the forms of the output results, the experimental data of 2024-T351 aluminum alloy [42] with different stress ratios, as shown in Figure 4, are employed as an example. The experimental information is listed in Table 1. In Figure 4, the *x*-axis and *y*-axis are the stress intensity factor range and fatigue crack growth rate, respectively. The different markers represent different stress ratios. It can be observed that the experimental data with different stress ratios show obvious nonlinear tendencies even in the Paris region.

The preprocessed ΔK and *R* are used to train the MLAs and output the da/dN following the procedures in Figure 3. MLAs will deeply learn the internal relationships from the limited fatigue crack growth data and establish a new continuous function between the driving force parameters and da/dN, which can be expressed as

(3) $$da/dN=f_{MLA}\left(\Delta K,\;R\right),$$

**Figure 5:**
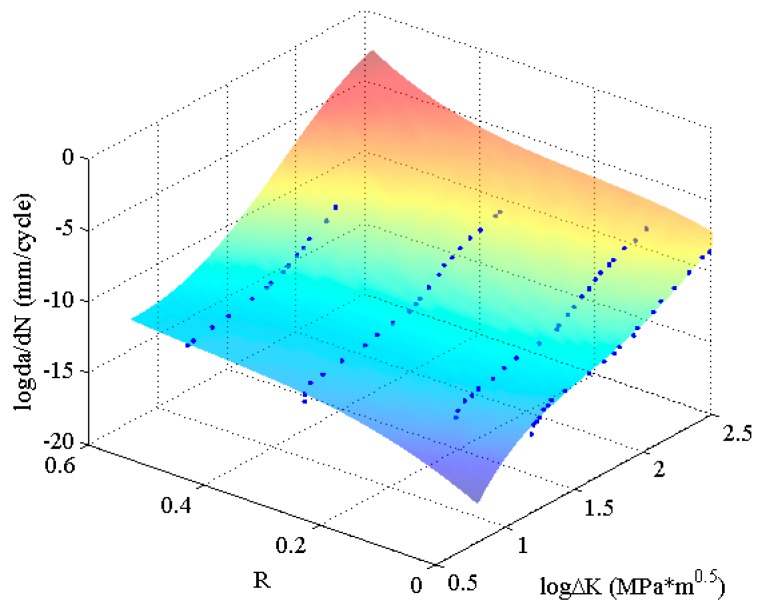
The predicted surfaces by MLAs and training data of 2024-T351 aluminum alloy.

As the experimental data are discrete, they cannot be used to calculate the fatigue crack growth rate. However, the continuous function is suitable for fatigue life prediction in mathematic approach. Furthermore, the continuous function can be shown as a smooth surface by interpolating and extrapolating da/dN with different stress ratios as shown in Figure 5, where the blue dots are experimental data used for training MLAs. It can be seen that the predicted surface beautifully agree with the experimental data. Then the da/dN at arbitrary (ΔK, *R*) on the surface can be calculated by using the Equation (3). Additionally, the tendency of the surface along a certain *R* is nonlinear, which is more consistent with the experimental data than the results of classical formula methods. Therefore, the MLAs have significant advantages in predicting and extrapolating the fatigue crack growth rate.

### 2.3. The Fatigue Life Prediction Method

After the MLAs are established and trained, the predicted results of MLAs will be used for fatigue life prediction in this section. The fatigue life prediction is based on the crack length increment (*da*) in one loading cycle. The basis theory is to calculate the crack length (a) at the Nth cycle by accumulating the increments of each loading cycles to the initial crack length (*a*_0_). The equation can be expressed as

(4) $$a_{N}=a_{0}+\overset{N}{\underset{i=1}{\sum }}da_{i},$$

where $a_{N}$ is the crack length at the Nth loading cycle, and $da_{i}$ is the increment in the ith cycle. In the process of fatigue crack growth, the $da_{i}$ is the da/dN in the ith loading cycle, which can be computed by MLAs as

(5) $$da_{i}={\left(da/dN\right)}_{i}=f_{MLA}\left(\Delta K_{i},R\right),$$

where $f_{MLA}\left(\Delta K_{i},R\right)$ means the generalized function between driving force parameters and da/dN established by MLAs. The $\Delta K_{i}$ can be calculated by the following two equations:

(6) $$\Delta K_{i}=Y\Delta \sigma \sqrt{\pi a_{i}},$$

(7) $$Y=g\left(\frac{a_{i}}{w}\right),$$

where Δσ and Y are the stress amplitude and geometric factor, respectively. The Y can be calculated by using the corresponding function g which depends on the specimen types, where *w* is the width of the specimen. According to the above functions, the relationship between loading information and the crack increment in every cycle is established. The fatigue crack growth equation can be integrated as

(8) $$a_{N}=a_{0}+\overset{N}{\underset{i=1}{\sum }}f_{MLA}\left(\Delta K_{i},R\right),$$

After that, the fatigue life prediction method based on MLA has been developed, and the crack length at any cycle can be calculated by using Equation (8).

**Figure 6:**
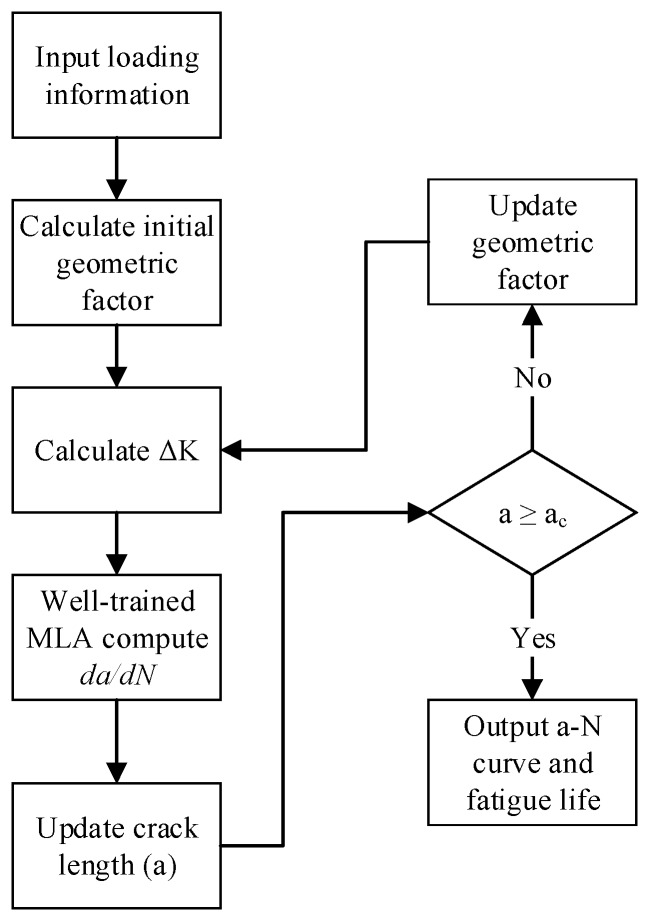
The flow chart of MLA based fatigue crack growth calculation method.

There are three main steps to calculate the fatigue crack growth using the MLA based method as shown in Figure 6. First, the initial geometric factor and ΔK must be calculated according to the loading information. Second, the well-trained MLA computes the da(da/dN) in current cycle to update the crack length. Third, the algorithms judge whether the crack length reaches the critical crack length (*a_c_*) and determine whether to update the geometric factor and ΔK for the next cycle. These three steps are iterated until the critical crack length is reached. Finally, the fatigue life curves (a-N curves) and the corresponding data are outputted.

## 3. Validation and Discussion

### 3.1. Fatigue Crack Growth Rate

A feasible theory and methodology for using the three MLAs for fatigue crack growth rate calculation has been introduced. In this section, various experimental data of different materials and characteristics are employed to validate the practical effectiveness of MLAs. The three MLAs are compared with each other to demonstrate the advantages and disadvantages. The training results of MLAs listed in this paper are the best choosing from a number of trial and error. Additionally, the predictions of classical $K^{*}$ approach are also utilized to compare with the results of MLAs with respect. The *α* value in $K^{*}$ formula of different materials references the results of Kujawski’s researches for $K^{*}$ approach [14,43].

**Figure 7:**
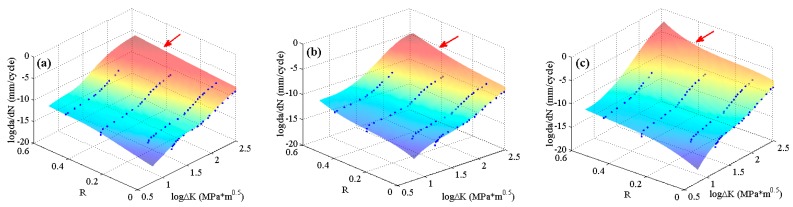
The predicted surfaces by MLAs and training data of 2024-T351 aluminum alloy: (**a**) genetic algorithms optimized back propagation (GABP); (**b**) radial basis function network (RBFN); and (**c**) extreme learning machine (ELM).

The experimental data of 2024-T351 aluminum alloy [42] with four stress ratios in the threshold region and the Paris region are employed to train the MLAs as shown in Figure 4. In the figure, it can be seen that the experimental data of 2024-T351 aluminum alloy show typically nonlinear characteristics even in the Paris region which is different from Paris’ law. The predicted results are shown in Figure 7. The three MLAs all can output the continuous and smooth surfaces, and all the surfaces show perfect nonlinearities which fit the experimental data very well as a whole. However, the three predicted surfaces have some differences on the tendencies in both the threshold region and the Paris region. The downward tendency of ELM is more obvious than that of RBFN and GABP in the threshold region. As the red arrows show in Figure 7, the tendency in the Paris region of ELM is upward, which is more consistent with the experiments, while that of RBFN is almost flat and the GABP is a little downward.

**Figure 8:**
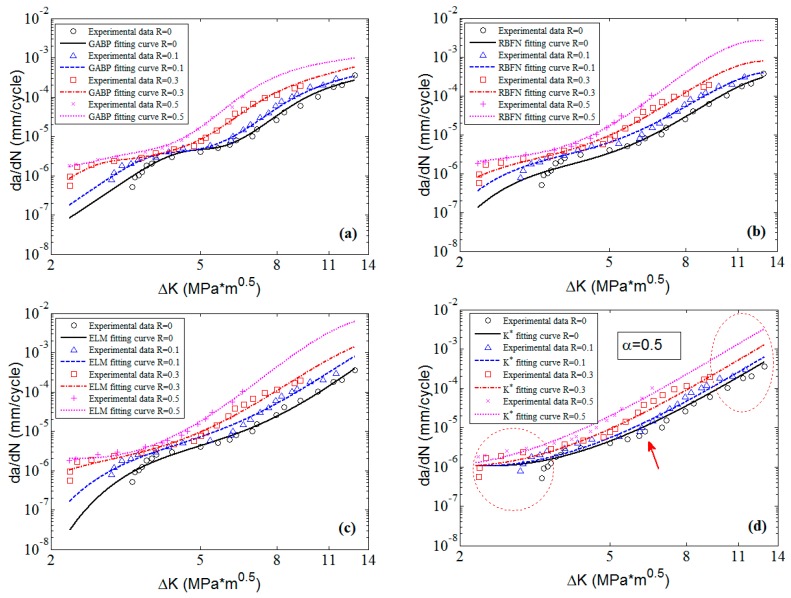
The predicted curves by MLAs and $K^{*}$ approach with experimental data of 2024-T351 aluminum alloy: (**a**) GABP; (**b**) RBFN; (**c**) ELM; and (**d**) $K^{*}$ approach.

To clearly check the effectiveness and accuracy of the predicted results, Figure 8 shows the predicted curves and the corresponding experimental data in 2D graphs. It is obvious that the predicted curves of the three MLAs all can perfectly fit the nonlinearities of the experimental data as a whole, and the performance of MLAs are much better than that of $K^{*}$ approach in nonlinearity and accuracy, especially in the threshold region as the red circle shows in Figure 8. Furthermore, in the Paris region, the MLAs match the experimental data with more excellent nonlinearity than $K^{*}$ approach. However, the MLAs show different performance in dealing with some details. In the Paris region, though the three MLAs all fit the experimental data very well, the GABP is better than the RBFN and the ELM as the GABP can fit any nonlinear characteristics of the experimental data, while the other two MLAs are meet with the global tendencies. Actually, in the threshold region, the da/dN will quickly decrease with the ΔK closing to the threshold value. In the end of the Paris region, the da/dN will keep the stable increasing tendency, similar to the whole Paris region. It is obvious that the ELM performs best in handling both tendencies. In other words, the ELM can fit the nonlinear characteristics of the experimental data by globally learning and optimizing the nonlinearities while the other two MLAs may be affected by some of the data points in the both ends of the curves.

**Figure 9:**
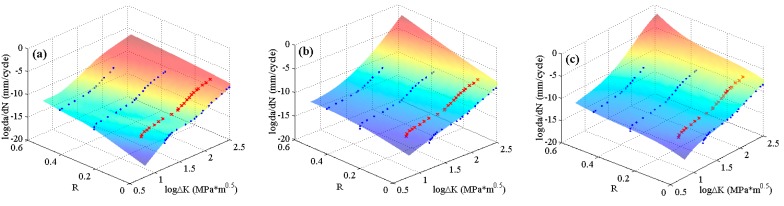
The predicted surfaces of MLAs trained with part of data: (**a**) GABP; (**b**) RBFN; and (**c**) ELM.

In this part, the cross-validation is conducted to investigate the predictive capacity of the MLAs by using the same experimental data of 2024-T351 aluminum alloy [42]. This time only three sets of the experimental data with stress ratios 0, 0.3 and 0.5 are utilized to train the MLAs. The experimental data with stress ratio 0.1 are used for validation. The predicted surfaces and experimental data are shown in Figure 9. The blue dots are the training data, and the red crosses represent the validation data. It can be seen that the accuracy is still satisfactory compared with the predictions in Figure 7. The predicted MSEs of the GABP, RBFN and ELM are 3.07 × 10^−7^, 9.91 × 10^−8^ and 1.33 × 10^−8^, respectively.

**Figure 10:**
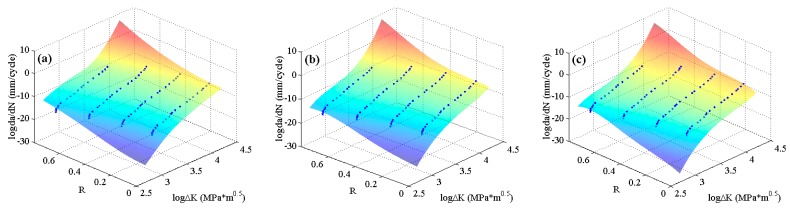
The predicted surfaces by MLAs and training data of 6013-T651 aluminum alloy: (**a**) GABP; (**b**) RBFN; and (**c**) ELM.

To further validate the globally optimizing characteristics of the MLAs, the experimental data of 6013-T651 aluminum alloy [5], which include all three regions of da/dN−ΔK curves, are employed. The predicted surfaces are shown in Figure 10. It can be seen that there is no obvious distinction for the surfaces predicted by the three MLAs, all of which describe the crack growth rate variation under different stress ratios very well.

**Figure 11:**
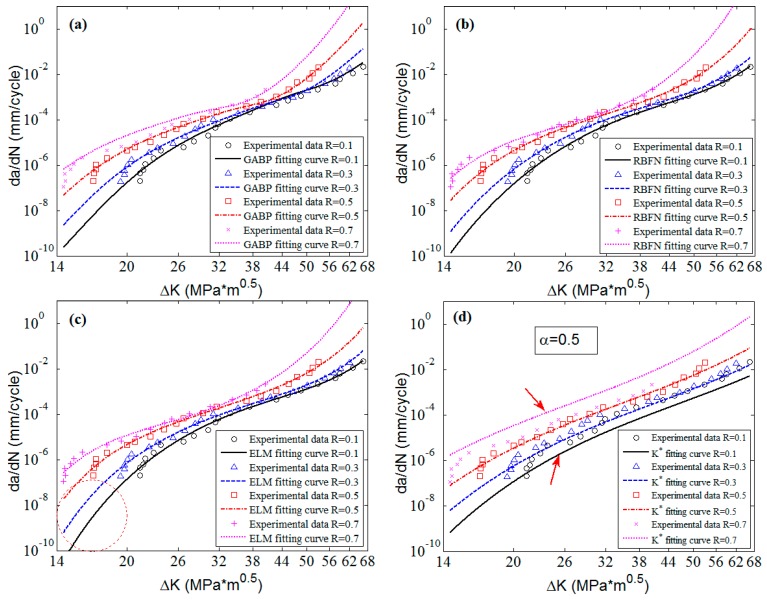
The predicted curves by MLAs and $K^{*}$ approach with experimental data of 6013-T651 aluminum alloy: (**a**) GABP; (**b**) RBFN; (**c**) ELM; and (**d**) $K^{*}$ approach.

The corresponding 2D graphs of 6013-T651 aluminum alloy with different stress ratios are plotted in Figure 11. It is clear that the predicted curves of the three MLAs match the experimental data of each stress ratio with perfect nonlinearity. However, the fitting curves of the $K^{*}$ approach are approximate “parallel linear” which cannot fit the nonlinearities of the experimental data, especially when the stress ratios are 0.1 and 0.7. It indicates that the stability of MLAs are better than that of the $K^{*}$ approach. Though the three MLAs can all fit the experimental data in the Paris region, the ELM can fit the fast change tendencies of da/dN in the threshold region and the high ΔK region better than the GABP, and the RBFN is almost comparable with the ELM. Additionally, the ELM extrapolates the nonlinearities of the experimental data better than the GABP and the RBFN when the predicted curves are beyond the ΔK ranges of experimental data as the red circle shows in Figure 11. That is to say, the excellent generalization performance of ELM means it can globally fit the nonlinearities of the experimental data, which is superior to the RBFN and the GABP.

**Figure 12:**
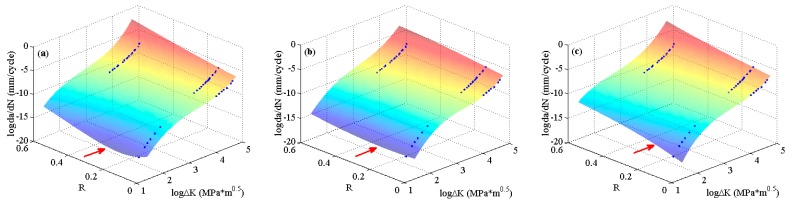
The predicted surfaces by MLAs and training data of 4340 steel: (**a**) GABP; (**b**) RBFN; and (**c**) ELM.

In order to more clearly contrast the extrapolating performance of each MLA, the experimental data of 4340 steel [44] with narrow ΔK ranges for each stress ratios are employed. The predicted surfaces are shown in Figure 12. The results reveal that the three MLAs can output satisfactory surfaces by using the small size experimental data without considering the accuracy and effectiveness. However, the tendencies of the predicted surfaces are different in the threshold region as the red arrows show. It can be seen that the ELM can extrapolate the nonlinearities better than the RBFN and the GABP.

**Figure 13:**
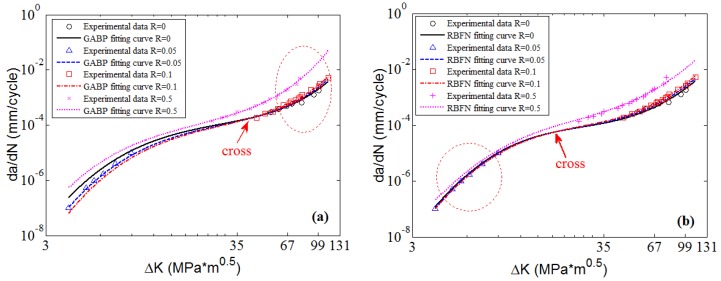

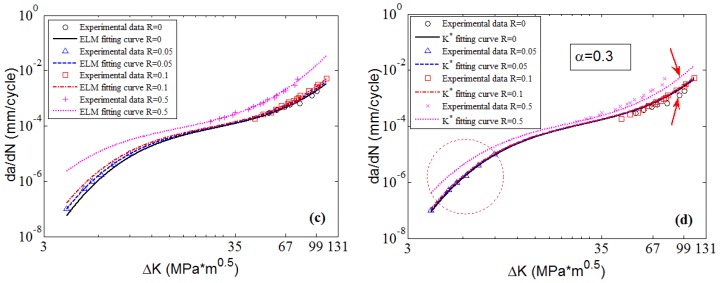
The predicted curves by MLAs and $K^{*}$ approach with experimental data of 4340 steel: (**a**) GABP; (**b**) RBFN; (**c**) ELM; and (**d**) $K^{*}$ approach.

To validate the accuracy and effectiveness of the three MLAs, the predicted curves and experimental data of 4340 steel in 2D graphs are shown in Figure 13. Since it is not easy to distinguish the goodness-of-fit of the three MLAs visually, the MSEs of the three MLAs’ fittings are given. The MSEs of the GABP, RBFN and ELM are 7.32 × 10^−5^, 8.89 × 10^−8^ and 3.12 × 10^−8^, respectively, which indicates that the three MLAs all can match the experimental data very well, and the ELM is the best one. The $K^{*}$ approach cannot fit the experimental data when the stress ratios are 0 and 0.5, which indicates that the three MLAs are more accurate than $K^{*}$ approach. However, the effectiveness of the three MLAs are totally different. When the ΔK is in the range where there are many training data with different stress ratios, as the red ellipse shows in Figure 13, the tendencies of the predicted curves are almost the same, which fit the experimental data very well. When the ΔK is in the extrapolating range where there is no or a few training data with different stress ratios as the red circles show in Figure 13, the predicted curves of MLAs are quite different. The GABP curves of *R* = 0 and 0.1 cross with each other and the RBFN curves of *R* = 0, 0.05 and 0.1 almost converge to one in the extrapolating range, which is impossible in experiments for 4340 steel. Therefore, ELM is the only one of the three MLAs not only can excellently fit the experimental data, but also extrapolate the nonlinear tendencies very well. In other words, when dealing with small size fatigue crack growth data such as for 4340 steel, ELM is the best one, although all the three MLAs can learn and predict the nonlinearities of experimental data very well.

**Figure 14:**
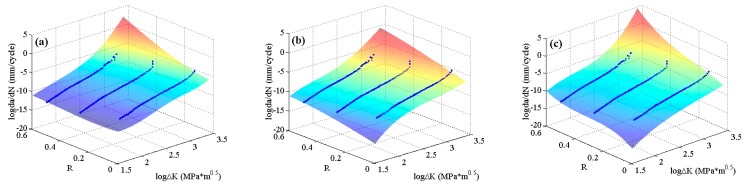
The predicted surfaces by MLAs and training data of 7050-T7451 aluminum alloy: (**a**) GABP; (**b**) RBFN; and (**c**) ELM.

**Figure 15:**
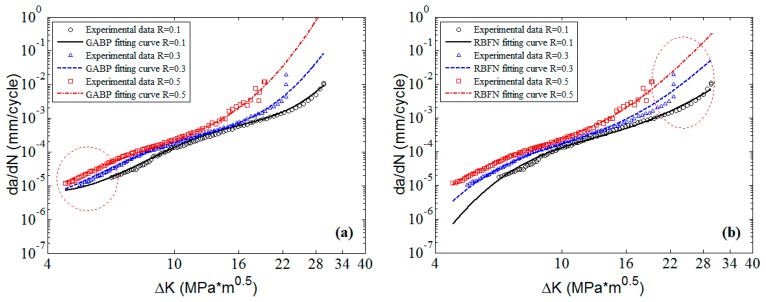

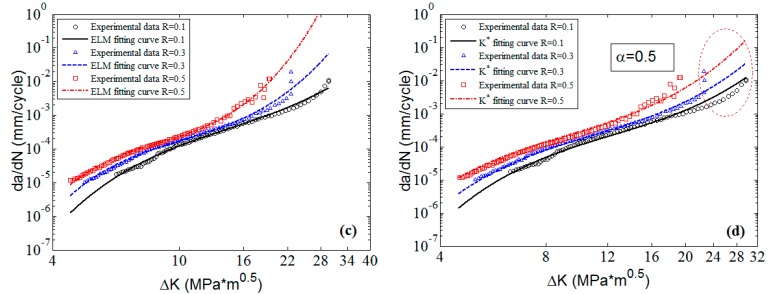
The predicted curves by MLAs and $K^{*}$ approach with experimental data of 7050-T7451 aluminum alloy: (**a**) GABP; (**b**) RBFN; (**c**) ELM; and (**d**) $K^{*}$ approach.

**Figure 16:**
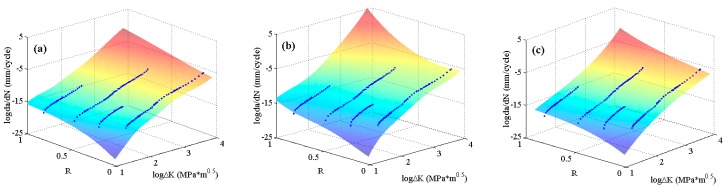
The predicted surfaces by MLAs and training data of Ti6Al4V titanium alloy: (**a**) GABP; (**b**) RBFN; and (**c**) ELM.

**Figure 17:**
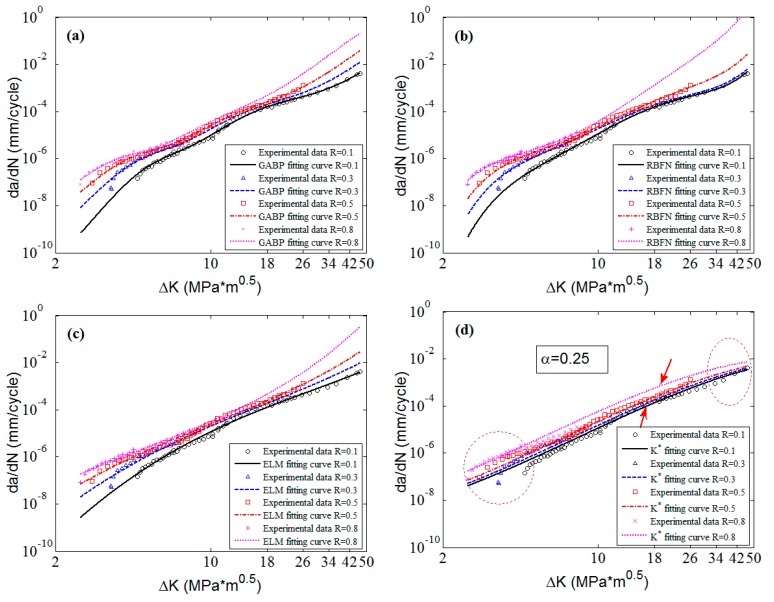
The predicted curves by MLAs and $K^{*}$ approach with experimental data of Ti6Al4V titanium alloy: (**a**) GABP; (**b**) RBFN; (**c**) ELM; and (**d**) $K^{*}$ approach.

**Figure 18:**
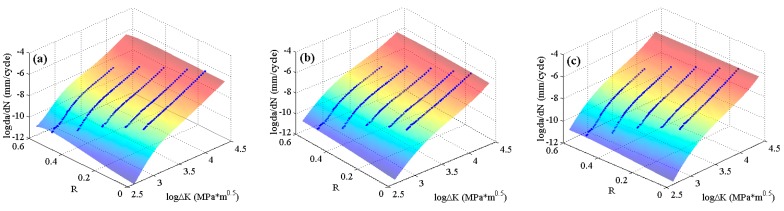
The predicted surfaces by MLAs and training data of ADB610 steel: (**a**) GABP; (**b**) RBFN; and (**c**) ELM.

**Figure 19:**
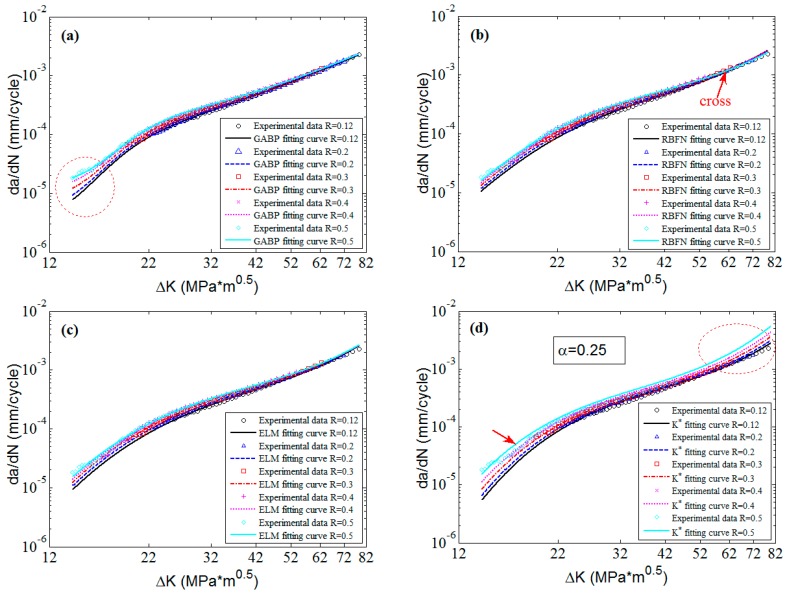
The predicted curves by MLAs and $K^{*}$ approach with experimental data of ADB610 steel: (**a**) GABP; (**b**) RBFN; (**c**) ELM; and (**d**) $K^{*}$ approach.

**Figure 20:**
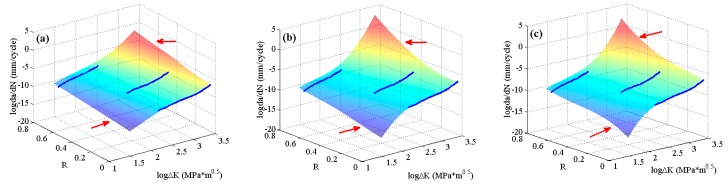
The predicted surfaces by MLAs and training data of D16 aluminum alloy: (**a**) GABP; (**b**) RBFN; and (**c**) ELM.

**Figure 21:**
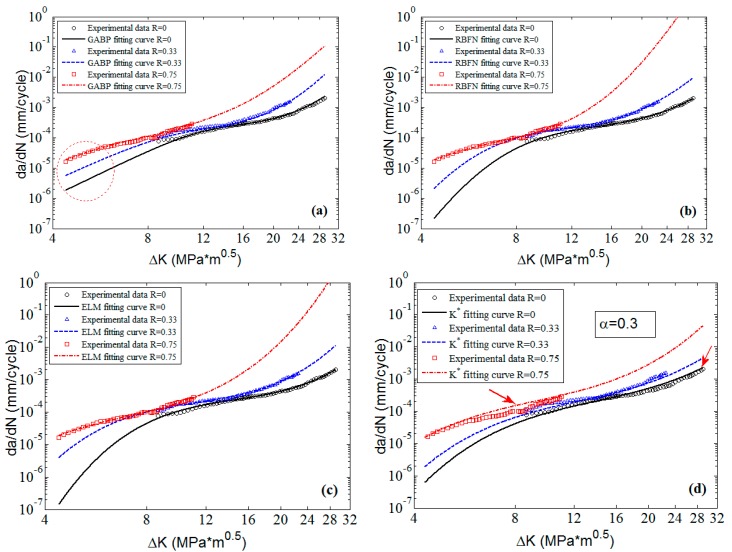
The predicted curves by MLAs and $K^{*}$ approach with experimental data of D16 aluminum alloy: (**a**) GABP; (**b**) RBFN; (**c**) ELM; and (**d**) $K^{*}$ approach.

In the following part, the flexibility and applicability of the three MLAs are further validated and compared with $K^{*}$ approach by using different kinds of materials including 7050-T7451 aluminum alloy, Ti6Al4V titanium alloy [45], ADB610 steel [7] and D16 aluminum alloy [46]. The predicted surfaces and 2D graphs are shown in Figure 14, Figure 15, Figure 16, Figure 17, Figure 18, Figure 19, Figure 20 and Figure 21, respectively. The results reveal that though the employed materials are of different fatigue crack growth characteristics, the three MLAs all can fit the experimental data very well with excellent nonlinearities, which is better than that of $K^{*}$ approach. Besides, the effectiveness and accuracy of MLAs are not affected by materials, which is superior to classical formula methods as the red arrows show in Figure 14, Figure 15, Figure 16, Figure 17, Figure 18, Figure 19, Figure 20 and Figure 21. Considering the extrapolating ability in the threshold region and the high ΔK region as the red circles and ellipses show in Figure 14, Figure 15, Figure 16, Figure 17, Figure 18, Figure 19, Figure 20 and Figure 21, the results of ELM are better than those of GABP and RBFN.

A comparison study of the three MLAs using different kinds of fatigue crack growth rate data indicates that it is feasible to apply the MLAs to fatigue crack growth calculation. The accuracy and effectiveness of MLAs compared well to those of $K^{*}$ approach. Additionally, the MLAs overcome the disadvantages of classical models (such as $K^{*}$ approach) that the applicability are affected by material parameters, such as $\Delta K_{th}$ and $K_{c}$. Therefore, the MLAs can be applied to a wider scope of materials, and the nonlinearities of MLAs are superior to those of $K^{*}$ approach. However, the three MLAs have their own advantages and limitations when used for fatigue crack growth calculation. The detailed comparisons based on the training results are summarized in Table 2. As a whole, the ELM is the best one used for fatigue crack growth calculation for its outstanding global optimization and generalization performance. Meanwhile, the ELM is the most convenient method to be trained, because it just have to tune the hidden neurons whose values are integer, while the adjustment parameters of GABP and RBFN can be more or any values which are hard to trial and error to find out the optimal combination. Moreover, when handling fatigue problems with less experimental data or stress ratios, such as 4340 steel, 7050-T7451 and D16 aluminum alloy in this paper, the GABP and the RBFN will be much more difficult and time-consuming than the ELM to obtain the satisfactory target outputs, especially the GABP whose results are not so good.

### 3.2. Fatigue Life Prediction

In order to validate the MLA based fatigue life prediction method, experimental data of 7050-T7451 aluminum alloy are employed. The experimental information is listed in Table 3. The well trained MLAs with the da/dN−ΔK data, as shown in Figure 14 and Figure 15, will be used to predict the fatigue life (a-N curves). Additionally, the classical $K^{*}$ approach will be also used to compare with the MLAs.

**Figure 22:**
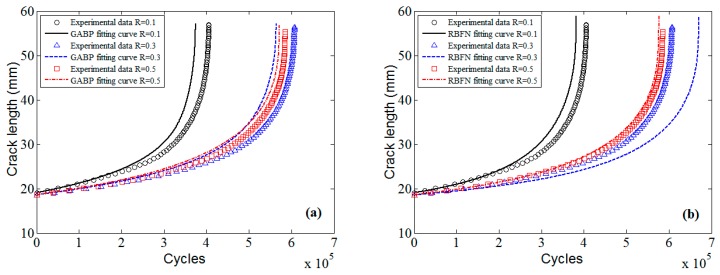

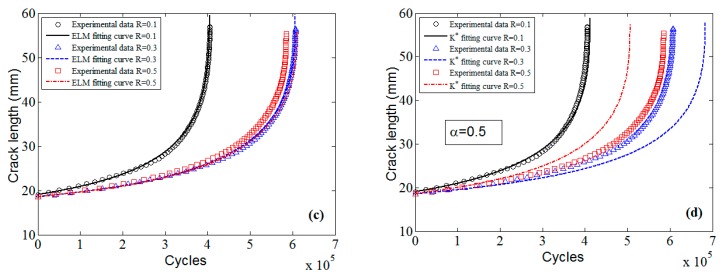
The predicted fatigue life and experimental data of 7050-T7451 aluminum alloy: (**a**) GABP; (**b**) RBFN; (**c**) ELM; and (**d**) $K^{*}$ approach.

The predicted a-N curves of 7050-T7451 aluminum alloy by MLAs and $K^{*}$ approach are displayed in Figure 22. The *x*-axis is the cycle number, and the *y*-axis is the crack length. By comparing to the experimental data, it can be seen that the predictions of MLAs are superior to those of $K^{*}$ approach, and the predictions of ELM are much better than those of GABP and RBFN.

The fatigue life can be calculated once the failure criterion is determined by using the predictions. To compare the prediction accuracy of MLAs with that of$\;K^{*}$ approach, the errors of the four methods when the critical crack length $\left(a_{c}\right)$ is 35 mm, 45 mm and 55 mm are listed in Table 4, where the positive value means that the predicted fatigue life is more than the experimental life, and the negative value represents that the predicted fatigue life is less than the experimental life. It is obvious that the accuracy of MLAs is globally much better than that of $K^{*}$ approach, especially the ELM whose accuracy and stability is the best.

To validate the flexibility and applicability of MLAs to different materials, ADB610 steel [7] is used to predict the fatigue life by using the well trained MLAs shown in Figure 18 and Figure 19. The results are compared with the classical $K^{*}$ approach. The experimental information of ADB610 steel is listed in Table 5.

**Figure 23:**
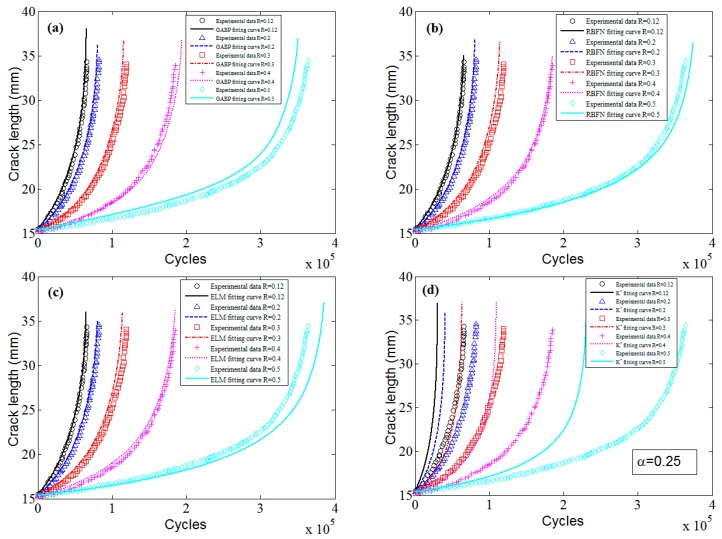
The predicted fatigue life and experimental data of ADB610 steel: (**a**) GABP; (**b**) RBFN; (**c**) ELM; and (**d**) $K^{*}$ approach.

The predicted a-N curves of ADB610 steel are shown in Figure 23. It can be seen that the predictions of MLAs are much better than those of the $K^{*}$ approach, and the three MLAs all show good accuracy.

The prediction errors of ADB610 steel when the critical crack length is 25 mm and 32 mm are listed in Table 6. The results show that the MLAs are better than $K^{*}$ approach in global accuracy and stability.

Furthermore, different experimental data of D16 aluminum alloy [46] are also used for model validation. The experimental information is listed in Table 7. Similarly, the surfaces and da/dN−ΔK curves predicted by the MLAs shown in Figure 20 and Figure 21 are utilized for fatigue life prediction, and the $K^{*}$ approach is also employed as a comparison.

**Figure 24:**
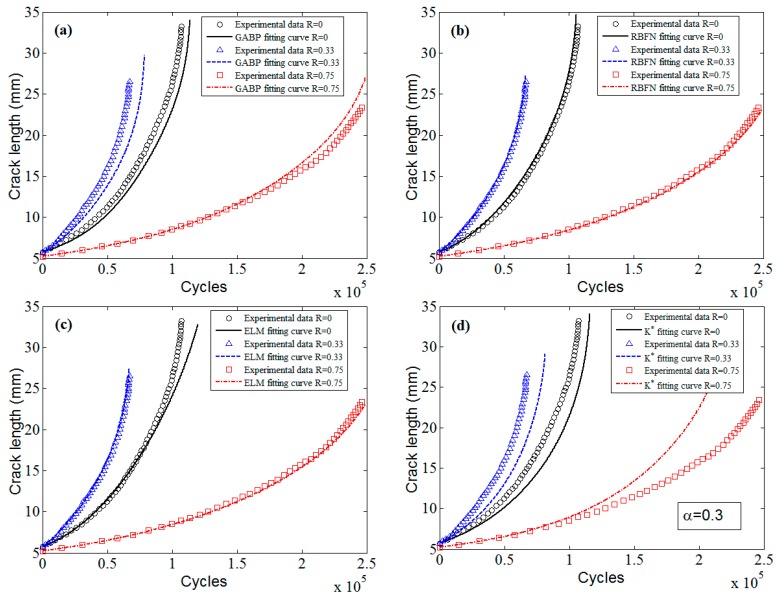
The predicted fatigue life and experimental data of D16 aluminum alloy: (**a**) GABP; (**b**) RBFN; (**c**) ELM; and (**d**) $K^{*}$ approach.

Figure 24 displays the predicted a-N curves of D16 aluminum alloy. It is obvious that the three MLAs all show pretty good performance, which are better than $K^{*}$ approach, especially the ELM and the RBFN.

The prediction errors of D16 aluminum alloy when the critical crack length is 15 mm, 18 mm and 22 mm are calculated in Table 8. The errors indicate the same conclusion that the MLAs are of excellent accuracy and stability, especially the ELM.

## 4. Conclusions and Future Work

In this paper, a fatigue crack growth calculation method based on MLA is proposed, and three MLAs are used for comparison study. The MLA based method is validated by using the testing data of different materials. The results indicate that the MLAs can fit the nonlinearities of fatigue crack growth rate very well, and the MLA based fatigue crack growth calculation method show fairly good performance for different experimental data. Additionally, the classical $K^{*}$ approach is also utilized to compare with the MLA based method. The comparison indicates that the MLA based method has higher applicability and accuracy than $K^{*}$ approach in all the examples, and the ELM based method is the best of the three MLAs for its excellent global optimization and extrapolation ability.

The above study demonstrates that the MLA based method has many advantages in handling the nonlinearities of fatigue crack growth. However, the performance of this method highly depends on the sufficiency of the experimental data, which are expected to include all the nonlinear information among all the possible ranges of ΔK and *R*. Therefore, the optimization of the training data combination need further study to guide the design of fatigue crack growth experiments, and then improve the MLAs’ efficiency and effectiveness.

## Figures and Tables

**Table 1:** The experimental information of 2024-T351 aluminum alloy.

Specimen Information		*R*	*σ*_min_	*σ*_max_
Specimen type	In-plane bending specimen	0	0 MPa	80MPa
Specimen size	52 × 7.2 × 3.2 mm (length × width × thickness)	0.1	9 MPa	86 MPa
Initial crack length	0.18–0.63 mm	0.3	23 MPa	76 MPa
Loading type	Constant amplitude	0.5	46 MPa	92 MPa

**Table 2:** Technique comparisons of MLAs used for fatigue crack growth.

MLAs	Advantages	Disadvantages
**GABP**	Strong local adjustment performance make it can fit any nonlinear variations Suitable for fatigue with plenty of data	Bad generalization performance Difficult for training as it has 6~10 adjustment parameters Consume long time to train (200~500 s)
**RBFN**	Can be automatically trained by using MATLAB toolbox Only one adjustment parameters (MSE goal) High training speed (0.3~0.6 s)	Too sensitive to MSE goal make it hard to trial and find out the optimal parameter Not suitable for small size fatigue data
**ELM**	Excellent global optimization and generalization performance Convenient to train for only one adjustment parameter (hidden layers) High training speed (0.1~0.2 s) Very suitable for small size fatigue data with different stress ratios	Cannot completely fit every nonlinear variations because of its global optimization performance

**Table 3:** The experimental information of 7050-T7451 aluminum alloy.

Specimen Information		*R*	*σ*_min_	*σ*_max_
Specimen type	Compact tensile specimen	0.1	0.53 MPa	5.28 MPa
Specimen size	100 × 80 × 8 mm (length × width × thickness)	0.3	1.64 MPa	5.47 MPa
Initial crack length	18.5 mm	0.5	3.44 MPa	6.88 MPa
Loading type	Tension-tension, constant amplitude	-	-	-

**Table 4:** The comparison of fatigue life errors for 7050-T7451 aluminum alloy.

*a*_c_	*R*	GABP	RBFN	ELM	K^*^ Approach
35 mm	0.1	−7.36%	−7.51%	−0.52%	0.47%
	0.3	−7.90%	11.14%	−0.83%	13.23%
	0.5	−3.73%	−1.90%	4.22%	−13.59%
45 mm	0.1	−7.83%	−6.09%	−0.16%	1.80%
	0.3	−7.35%	10.88%	−0.09%	12.94%
	0.5	−2.53%	−1.58%	4.13%	−13.72%
55 mm	0.1	−7.63%	−5.72%	−0.18%	1.66%
	0.3	−7.03%	10.50%	−0.08%	12.65%
	0.5	−2.30%	−1.45%	4.21%	−13.45%

**Table 5:** The experimental data of ADB610 steel.

Specimen Information		*R*	*σ*_min_	*σ*_max_
Specimen type	Compact tensile specimen	0.12	2.4 MPa	20 MPa
Specimen size	60 × 50 × 12.5 mm (length × width × thickness)	0.2	4 MPa	20 MPa
Initial crack length	15 mm	0.3	6 MPa	20 MPa
Loading type	Tension-tension, constant amplitude	0.4	8 MPa	20 MPa
-	-	0.5	10 MPa	20 MPa

**Table 6:** The comparison of fatigue life errors for ADB610 steel.

*a*_c_	*R*	GABP	RBFN	ELM	K^*^ Approach
25 mm	0.12	−3.91%	−2.82%	−3.44%	−51.92%
	0.2	−2.90%	−2.64%	−1.95%	−47.19%
	0.3	−3.64%	−4.83%	−4.60%	−43.67%
	0.4	3.87%	−0.99%	−1.68%	−38.48%
	0.5	−4.88%	2.31%	5.47%	−34.13%
32 mm	0.12	−1.93%	−0.65%	−1.32%	−37.98%
	0.2	−2.46%	−2.49%	−1.90%	−50.06%
	0.3	−3.98%	−5.45%	−5.19%	−46.80%
	0.4	3.96%	−0.32%	−0.87%	−39.85%
	0.5	−4.56%	2.29%	5.19%	−36.23%

**Table 7:** The experimental information of D16 aluminum alloy.

Specimen Information		*R*	*σ*_min_	*σ*_max_
Specimen type	Middle cracked tension specimen	0	0 MPa	64 MPa
Specimen size	500 × 100 × 0.04 mm (length × width × thickness)	0.33	32 MPa	96 MPa
Initial crack length	5 mm	0.75	105 MPa	140 MPa
Loading type	Tension-tension, constant amplitude	-	-	-

**Table 8:** The comparison of fatigue life errors for D16 aluminum alloy.

*a*_c_	*R*	GABP	RBFN	ELM	K^*^ Approach
15 mm	0	8.40%	−3.65%	−0.53%	15.26%
	0.33	15.24%	−2.17%	−1.97%	21.28%
	0.75	−2.91%	1.62%	1.97%	−15.55%
18 mm	0	7.67%	−2.51%	1.18%	14.01%
	0.33	15.61%	−1.29%	−1.28%	19.46%
	0.75	−6.89%	−2.15%	−2.01%	−20.10%
22 mm	0	7.39%	−1.66%	3.47%	11.90%
	0.33	14.74%	−1.64%	−1.65%	17.58%
	0.75	−3.39%	1.43%	1.47%	−17.66%
